# Selective and flexible depletion of problematic sequences from RNA-seq libraries at the cDNA stage

**DOI:** 10.1186/1471-2164-15-401

**Published:** 2014-05-26

**Authors:** Stuart K Archer, Nikolay E Shirokikh, Thomas Preiss

**Affiliations:** Genome Biology Department, The John Curtin School of Medical Research (JCSMR), The Australian National University, Acton, Canberra, Australian Capital Territory, Australia; Moscow Regional State Institute of Humanities and Social Studies, Ministry of Education of Moscow Region, Kolomna, Moscow Region Russia

**Keywords:** RNA-seq, rRNA, Library, cDNA, Duplex specific nuclease, Depletion, PDD

## Abstract

**Background:**

A major hurdle to transcriptome profiling by deep-sequencing technologies is that abundant transcripts, such as rRNAs, can overwhelm the libraries, severely reducing transcriptome-wide coverage. Methods for depletion of such unwanted sequences typically require treatment of RNA samples prior to library preparation, are costly and not suited to unusual species and applications. Here we describe Probe-Directed Degradation (PDD), an approach that employs hybridisation to DNA oligonucleotides at the single-stranded cDNA library stage and digestion with Duplex-Specific Nuclease (DSN).

**Results:**

Targeting Saccharomyces cerevisiae rRNA sequences in Illumina HiSeq libraries generated by the split adapter method we show that PDD results in efficient removal of rRNA. The probes generate extended zones of depletion as a function of library insert size and the requirements for DSN cleavage. Using intact total RNA as starting material, probes can be spaced at the minimum anticipated library size minus 20 nucleotides to achieve continuous depletion. No off-target bias is detectable when comparing PDD-treated with untreated libraries. We further provide a bioinformatics tool to design suitable PDD probe sets.

**Conclusion:**

We find that PDD is a rapid procedure that results in effective and specific depletion of unwanted sequences from deep-sequencing libraries. Because PDD acts at the cDNA stage, handling of fragile RNA samples can be minimised and it should further be feasible to remediate existing libraries. Importantly, PDD preserves the original RNA fragment boundaries as is required for nucleotide-resolution footprinting or base-cleavage studies. Finally, as PDD utilises unmodified DNA oligonucleotides it can provide a low-cost option for large-scale projects, or be flexibly customised to suit different depletion targets, sample types and organisms.

**Electronic supplementary material:**

The online version of this article (doi: 10.1186/1471-2164-15-401) contains supplementary material, which is available to authorized users.

## Background

The application of RNA-seq for in-depth transcriptomics analyses is hindered by the vast excess of certain RNAs leading to insufficient coverage of transcripts of interest. Such problematic sequences can be rRNA (or fragments thereof, e.g. as in degraded archival samples), RNAs from particular species (e.g. in environmental or host-pathogen transcriptomics) or canonical transcript sequences (e.g. in targeted sequencing to identify rare variants/modifications [1]). A particular challenge for experiments involving targeted isolation of complex-associated RNA, such as in ribosome profiling [2, 3], CLIP-seq [4, 5], or modified base cleavage studies is that the RNA must be partially fragmented, which releases a plethora of rRNA digestion products. This impedes interpretation of the results; for example in CLIP-seq, an input sample digested in parallel should be sequenced as a control to facilitate peak-calling from the sequence data [6], however this is rarely done in practice due to the high content of degraded rRNA in these controls.

Popular methods to deplete unwanted sequences involve hybridizing the RNA with biotinylated LNA probes (as in several commercial kits) or hybridizing the single-stranded cDNA library to biotinylated DNA probes [3], and depleting the bound targets using immobilized streptavidin. However, these probes are usually purchased as ready-made sets targeted at rRNA in a limited range of source species, precluding customization. The costs of obtaining custom modified probes to, for instance, target all rRNA fragments in degraded (e.g. archival samples) or digested samples (e.g. for CLIP-seq or ribosome profiling) are prohibitive. Targeting rRNA with unmodified antisense DNA oligonucleotides and digestion with RNase H can yield efficient depletion without introducing gross bias into the transcriptome [7, 8]. This approach, however, requires saturation of rRNA with contiguous oligonucleotides. Further, due to the high thermal stability of RNA:DNA hybrids [9] and the short duplex length (six base pairs) targeted by RNase H, there is no guarantee that the resulting fragment ends will all be faithful representations of the input fragment ends in nucleotide-resolution studies (particularly as RNase H yields polished, easily ligated RNA ends).

A strategy to deplete high-abundance sequences from dsDNA libraries is C_0_T-hybridization [10], which involves heat-denaturing followed by re-annealing. High-abundance sequences, which preferentially re-anneal, are removed using duplex-specific nuclease (DSN) [11] or hydroxyapatite chromatography [12]. This procedure has not been widely adopted due to the difficulty of fine-tuning the extended hybridization reaction. Application of C_0_T-hybridization to deep sequencing libraries is further severely limited by the potential of the ubiquitous linker/adapter sequences to be inadvertently targeted. Notably, DSN treatment in conjunction with targeted DNA oligonucleotide probes has been used to deplete abundant mRNA sequences in conventional, full-length cDNA libraries [13].

Here we outline probe-directed degradation (PDD), a DSN/probe hybridization-based method for depletion of unwanted cDNA sequences from RNA-seq libraries. Targeting *Saccharomyces cerevisiae* rRNA we achieved efficient depletion across multiple PDD-targeted loci without introducing bias among mRNA-derived sequences. We present probe design guidelines and a bioinformatics tool that evaluates the efficacy of candidate probe sets. We also describe some streamlining modifications to the split adapter library preparation strategy. Overall, we find PDD to be rapid and specific, while also being quickly and cheaply customizable to diverse species or applications.

## Methods

### Probe design

Probes were initially designed manually to target rRNA sequences that form the major contaminants in ribosome profiling libraries (data not shown). To enable *in silico* analysis of these probes for possible hybridization with other transcripts, we constructed a Perl script to identify matches of >10 nt in the transcribed portion of any annotated genome (in GenBank or Ensembl format), estimating the T_m_ of each target and off-target match by the method of Allawi *et al*.[14] as implemented in the BioPerl Primer module. Here we used the reference genome for *S. cerevisiae* S288C, assembly R64-1-1 (Saccharomyces Genome Database [15]). Probe sequences (Additional file 1: Table S1) were refined in several iterations to maximize the T_m_ differential between desired and undesired targets. They were then ordered in plate format from Integrated DNA Technologies and individually resuspended at 200 μM.

### Library preparation

A “degraded” RNA sample (used in qPCR analysis) was generated by incubating 1 AU_260_ of *S. cerevisiae* cytoplasmic lysate with 3 U of RNase 1 for 30 minutes at room temperature. A “total” RNA library (used for sequencing) was generated by Mg^+2^-mediated cleavage of intact RNA for 8 minutes at 94°C using the NEBNext Mg^+2^ RNA fragmentation module (New England Biolabs). A “RiboMinus™ spike-in” library (for assessing mRNA coverage and PCR duplication, was generated by mixing RiboMinus™-treated RNA (Life Technologies, generated as per manufacturer’s instructions) with total RNA in a 1:4 ratio and fragmenting for 6 minutes at 94°C. In both cases, libraries were generated by the split adapter method as described [2] with modifications (ExoI digestion and SPRI bead selection to rid the library of unextended RT primer, see below).

Library preparation was performed as follows. After ethanol precipitation and resuspension in T_10_E_0.1_, (10 mM Tris, 0.1 mM EDTA buffer pH 7.4) approximately 500 ng of fragmented RNA was end-repaired by 5 U of T4 Polynucleotide Kinase (wild-type, New England Biolabs) in 20 μl of 1× PNK buffer (no ATP) for 2 hours at 37°C in the presence of 1 U/μl of RNaseOUT inhibitor (Life Technologies). T4 PNK was then heat-inactivated at 65°C for 10 minutes and the resultant RNA was 3′ polyadenylated using the Ambion Poly(A)-tailing kit as follows. The PNK reaction was added to a master mix to give a final 50 μl reaction containing of 0.5 mM rATP, 2.5 mM MnCl_2_, 1× reaction buffer, 0.75 U of *E. coli* Poly(A) Polymerase and supplemented with fresh RNaseOUT RNAse inhibitor (Life Technologies). The polyadenylation reaction was allowed to proceed for 1 hour at 37°C. Pyrophosphate precipitate was pelleted by brief centrifugation and the RNA in the supernatant was ethanol-precipitated in a new tube. RNA pellets were resuspended in 15 μl of 1 mM sodium citrate buffer, pH 6.2. After polyadenylation, A_260_ readings were no longer useful for quantifying RNA.

Half of the resuspended RNA (7 μl, about 250 ng of the original RNA) was reverse transcribed using the SuperScript® III first-strand synthesis kit (Life Technologies) according to the manufacturer’s instructions with some variations as follows. 20 pmol of split adapter/oligo dT primer (Integrated DNA Technologies) were mixed with the template RNA and dNTPs in 13 μl and heated to 70°C for 3 minutes in a thermocycler, cooled to 60°C then slow-ramped to 55°C over 2 minutes to anneal primer. A preheated 7 μl reverse transcription master mix aliquot (containing buffer, SuperScript® III and RNaseOUT™) was added, the mixture was slow-ramped to 50°C and incubated for a further 30 minutes. The reverse transcription reaction was heated to 60°C for 5 minutes, then cooled to 37°C, and 1 μl of 20 U/μl ExoI (New England Biolabs) was immediately added, followed by a further 20 minute incubation to allow depletion of the unextended single-stranded primer. Meanwhile, AMPure XP beads (Beckman Coulter) from 20 μl of initial manufacturer’s suspension were washed and resuspended in 2.2× reaction volumes (44 μl) of 1× PN buffer (20% PEG-8000, 2.5 M NaCl). ExoI was inactivated by adding 1 μl 250 mM EDTA, and both the reaction mixture (22 μl) and the beads suspension (44 μl) were preheated in a 60°C oven before rapidly mixing and returning to the oven for 5 minutes. Beads were collected on a magnetic rack for 1 minute in the oven, the rack was transferred to room temperature and the supernatant was immediately removed and replaced with room-temperature 0.66× PN buffer while keeping the reaction tubes on the magnet. After two ~30 second washes in 70% ethanol on the magnet at room temperature, beads were dried ~2 minutes and cDNA was eluted in 13.3 μl T_10_E_0.1_ buffer. The purpose of the elevated temperature during bead binding was to prevent non-specific primer annealing and carryover, while still leaving cDNA:RNA duplexes intact to take advantage of the large length differential between the polyadenylated RNA and the primer for size-based selection on Ampure XP beads. We have not tested room-temperature separation conditions.

### cDNA circularization

Purified cDNA:RNA duplexes from the previous step were denatured at 80°C for 15 minutes and the RNA degraded by adding 0.7 μg of RNase A and incubating at 37°C for 20 minutes. An aliquot (9 μl) was circularised with 75 U of CircLigase™ II (Epicentre) in a 20 μl self-ligation reaction containing 1× manufacturer’s reaction buffer supplemented with 2.5 mM MnCl_2_ and 0.5 M betaine for 2–6 hours at 60°C. Upon completion of the circularisation reaction, EDTA was added to 2.5 mM and the ligase was heat-inactivated at 80°C for 15 minutes. The amount of cDNA product in the reaction was that derived from approximately 112 ng of starting RNA (however, we have successfully used as little as 90 ng in a 20 μl circularisation reaction). Circular cDNA was then purified using AMPure XP beads (using 20 μl of the initial bead mixture, washed and resuspended in 2× sample volumes of PN buffer at ambient temperature, and proceeding with binding and washing as per the manufacturer’s instructions) and the resultant bound cDNA library was eluted from the beads in 10 μl T_10_E_0.1_ buffer.

### DD treatment

Half of the circularised library (5 μl) was mixed with 2 μl of 3.5× DSN buffer (Evrogen) with depletion probes (50 probes at 0.81 μM each) so that each probe was approximately equimolar with the library assuming 100% conversion of RNA to cDNA. The 7 μl mix was overlayed with mineral oil and denatured on a thermocycler at 95°C for 1 minute, brought to 75°C and then slowly cooled (3°C/minute) to 48°C. After a 5 minute hybridization at 48°C, 3 μl of pre-warmed DSN master mix containing 0.4 U DSN (Evrogen) in 1× DSN buffer was added and incubated for 20 minutes. The reaction was stopped by the addition of 6 μl of 25 mM EDTA and incubated for another 5 minutes, then phenol:chloroform extracted and the DNA was purified using AMPure XP beads (at ambient temperature as described above) and the libraries eluted in 10 μl T_10_E_0.1_ each. The remaining half of the circularised library was left untreated as a control.

### Library amplification and size-selection

The libraries were amplified by PCR using indexed primers, corresponding to those used in the Illumina’s TruSeq Small RNA Sample Prep Kit. One third of each of the PDD-treated and untreated library samples from the previous step were added as templates to PCR mixtures (Platinum® *Pfx* DNA Polymerase supplemented with 1× buffer (Life Technologies), dNTPs, primers, and Extreme Thermophilic Single-Stranded Binding Protein (New England Biolabs)) and amplified through 15 thermocycles. Primers contained all the flanking sequences features necessary for sequencing on the intended platform, and different index sequences for each library. PCR products were run on a TBE DNA gel (Novex) and a band of ~170-350 bp (corresponding to insert sizes of 25–200 nt) was cut out, DNA from it was purified and analysed by on-chip electrophoresis using an Agilent Bioanalyzer. Libraries that were to be directly compared with each other (e.g. paired PDD-treated and untreated samples) were quantified and pooled together proportionally prior to size-selection on the gel. The indexed, size-selected libraries were pooled with other indexed size-selected libraries for sequencing from the 5′ ends (150 nt reads) on an Illumina HiSeq 2500 instrument by the Biomolecular Resource Facility, Australian National University.

### Sequence data analysis

Raw reads were trimmed for low-quality positions (Phred > 28) using fastq_quality_trimmer and adapter sequences and poly(A) tracts were then trimmed using fastx_clipper, both from the fastx tools suite. Reads shorter than 11 nt after trimming were discarded. Reads were initially mapped unidirectionally using BowTie2 [16] (default settings) against processed rRNA sequences, and the unmapped reads were then mapped against other non-coding RNAs including rRNA precursors, snoRNAs and tRNAs. Finally, the remaining unmapped reads were mapped unidirectionally against mRNAs (all predicted spliced ORFs). Reference sequences were from the S288C reference genome (assembly R64-1-1, Saccharomyces Genome Database [15]). Read densities and counts per transcript were collated using custom Perl and R scripts. Reads were first divided into size ranges, and the density of the 5′ ends of reads mapping to rRNA was calculated as a moving average (window size 9 nt) from PDD-treated and untreated libraries. Read 5′ density from PDD-treated libraries was plotted as a percentage of that from untreated libraries after normalizing for library loading using the total number of mRNA-mapped reads within the relevant insert size-group. Depletion efficiency was calculated from the minima of this value after averaging across all functional 18S and 25S probes.

GC content of libraries was calculated for all reads mapping to mRNAs from each library. PCR duplication rates were determined by analysing the number of reads starting at exactly the same position. Coverage was calculated as the number of reads divided by the number of available positions (estimated as the spliced ORF length minus the median read length) on each ORF. Simulated reshuffling of read 5′ ends was performed repeatedly over the available positions in each ORF and the number of exact 5′ read end overlaps was tallied and compared to the numbers from the real data (Additional file 2: Figure S2). At least 100 simulations were performed for ORFs with >10 reads, and at least 500 each for ORFs with 2–10 reads.

### qPCR

qPCR was performed using Quantifast qPCR premix (Qiagen) as per manufacturer’s instructions with primers as listed in (Additional file 1: Table S1) at 0.5 μM final concentration. Serial dilutions of control cDNA were used to test the PCR efficiency, which was >90% for all primers. 10 μl reactions were generated in 384-well plates (3 technical replicates per condition) and qPCR performed on a QuantStudio 12 K Flex (Invitrogen).

## Results

**Figure 1:**
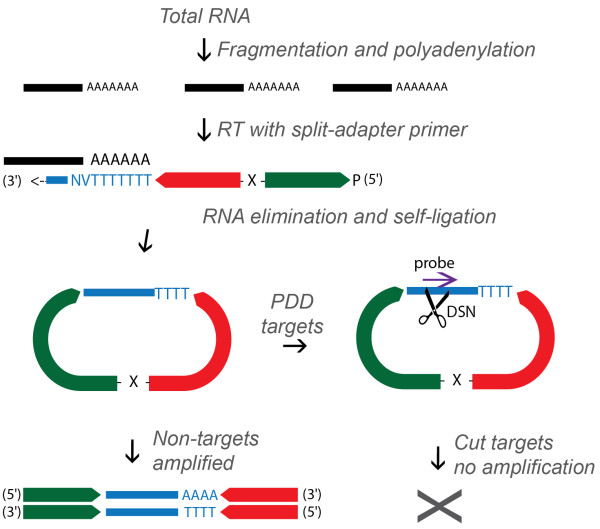
**Strategy to implement PDD with the split-adapter library preparation method**. Fragmented RNA is 3′-polyadenylated and reverse transcribed using a primer with both 5′ (green) and 3′ (red) adapters. Between them (‘X’) is a block for DNA polymerases (here, a C9 abasic spacer). Also present is a 5′-phosphate (P) for subsequent self-ligation. After reverse transcription, RNA template is eliminated and cDNA (blue) circularized and subjected to PDD treatment, whereby it is hybridized to DNA oligonucleotide probes (purple arrow) and any dsDNA formed is cut with DSN, rendering the targets incapable of amplification.

In developing PDD we aimed for a method that would avoid manipulations at the RNA level and use unmodified DNA oligonucleotide probes to allow for cost savings as well as facile customisation. These criteria were met by deploying probe-mediated DSN cleavage at the stage of single-stranded adapter-flanked cDNA, common to several RNA-seq library preparation methods, including the split adapter ligation approach used here (Figure 1) [2]. A single cut introduced anywhere in a library molecule will prevent its amplification via the adapter sequences at subsequent stages. This strategy also avoids inadvertent cleavage of adapter sequences, as there is no complementary strand with which they could form dsDNA. DSN operates efficiently at higher temperatures and requires at least ten perfectly complementary base pairs to cut, thus it has better mismatch discrimination than RNase H, especially given the higher T_m_ of RNA:DNA compared to DNA:DNA hybrids. That PDD is performed downstream of adapter ligation furthermore eliminates any risk of new adapter-insert junctions being created through off-target cutting.

### Proof of concept

**Figure 2:**
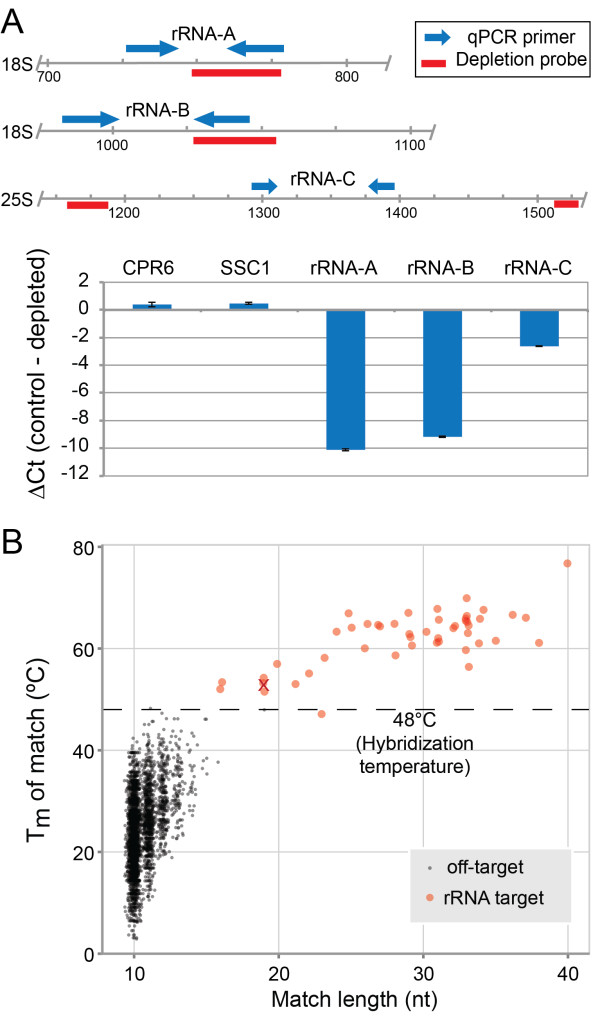
**Initial PDD trials and probe set analysis. A**: Top: Two qPCR primer pairs (red arrows), rRNA-A and rRNA-B, flanked the target sites for depletion probes (blue bars), while the rRNA-C primer pair was located about 100 nt away from the nearest target sites. Bottom: qPCR ΔCt values (untreated relative to PDD-treated libraries) from the three rRNA sequences and two mRNA controls. Error bars are the standard error of the mean. **B**: Fifty depletion probes were analysed for T_m_ of hybridization with intended rRNA targets (black) and likely off-target sites (red) in the transcriptome using OffTarget_Tm. The probe that later failed to deplete its target is indicated (red cross).

To test the feasibility of the PDD strategy, we simulated the generation of a RNA-seq library from degraded RNA by using RNase I-treated *S. cerevisiae* cytoplasmic lysate as starting material for the split-adapter library preparation method [2]. This method, which has been extensively characterized elsewhere [17, 18] and is now also implemented as a commercial kit, was chosen due to its even coverage and low tendency for insert bias. It generates a single-stranded intermediate antisense cDNA construct flanked by 5′ and 3′ adapters in a circular molecule (Figure 1). We mixed the library intermediate with four oligonucleotide probes corresponding to rRNA sense sequences (Figure 2A, top), denatured and cooled to 48°C, whereupon DSN was added to one half of the mixture and the other mock incubated. After library amplification, qPCR was performed for several rRNA amplicons as well as mRNA sequences for normalisation purposes. This showed that the abundance of the two directly targeted sequences (rRNA-A and rRNA-B) had decreased by approximately two orders of magnitude in the DSN-treated library, while another qPCR amplicon (rRNA-C), ~100 nt away from two flanking probe-targeted sequences, still decreased by about 5-fold (Figure 2A, bottom).

### Probe design

Following these encouraging findings, we designed more sense-strand DNA oligonucleotide probes to target rRNA fragments that tend to dominate ribosome profiling libraries to reach a total of 24 each, against 18S and 25S rRNA, and one each, against 5.8S and 5S rRNA (see Additional file 1: Table S1). The probes had variable spacing across the rRNAs, allowing characterisation of individual probe efficacy and probe spacing requirements. We also generated a bioinformatics tool, OffTarget_Tm (Additional file 3), to evaluate probe sets. OffTarget_Tm performs *in silico* annealing of probes to the target RNAs and other transcriptome sequences in order to identify potential unintentional off-targets and determine the optimum hybridization temperature for PDD. Results with our probe set indicated excellent discrimination between intended targets and off-targets at our chosen hybridization temperature of 48°C (dashed line, Figure 2B).

### Application to RNA-seq libraries

To demonstrate the performance of PDD in full-scale RNA-seq experiments, we generated two high-complexity RNA-seq libraries by the split adapter method, this time using Mg^+2^–mediated fragmentation of purified *S. cerevisiae* RNA. One library was made from highly fragmented total RNA (8 minute treatment). The other library was made from a moderately fragmented (6 minute treatment) mixture of total RNA spiked with 20% of RNA that had been enriched for mRNA using the RiboMinus™ kit. The purpose of the first library was to assess PDD efficacy in a typical application. The goal with the second library was to have better mRNA representation (particularly for the untreated control sample) to accurately assess any off-target effects of PDD. We also modified the published split adapter ligation protocol (see Methods) to include selection of cDNA:RNA duplexes away from unextended reverse-transcription primer (which can cause adapter dimers to form in subsequent steps), while bypassing several gel-electrophoresis purification steps.

Each cDNA library intermediate was split into equal aliquots and either PDD treated or left untreated. The four samples were then PCR-amplified, size-selected for an insert size of ~20 - 200 nt and the first 150 nt sequenced from the 5′ end in a multiplexed run on an Illumina HiSeq 2500 instrument.

### Efficient depletion of rRNA

**Figure 3:**
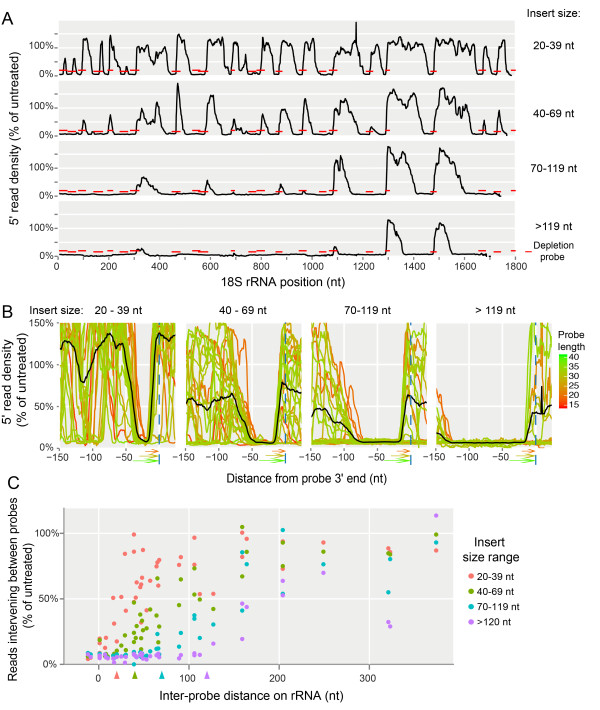
**PDD-mediated rRNA depletion. A**: 18S rRNA profile of read 5′ end-density (PDD-treated as a percentage of that of untreated) from the total RNA library. Reads were divided into the insert length ranges indicated on the right and normalized to the number of reads mapped to mRNA ORFs within each size category. Annealing positions of depletion probes are indicated (red bars). **B**: Pile-up of 5′ read densities (PDD-treated as a percentage of untreated) flanking all probes targeting 18S and 25S rRNA (moving average with 9 nt window, x-coordinate is relative to the probe 3′ end) in four different size-ranges of library inserts. Black lines: average read ratio of smoothed (moving average, 7 nt window) individual profiles. Probe lengths are indicated by line colours. **C**: Trade-off between library insert size and probe spacing from the total RNA library. For each pair of adjacent probes along rRNA, reads falling between probes (+10 nt extending into each probe) were counted, and used to calculate a normalized read ratio of each inter-probe segment of rRNA (PDD-treated as a percentage of untreated; *y*-axis). This was compared to the intervening distance between probes (*x*-axis, see Additional file 2: Figure S1B for a schematic). Four different library insert size ranges were analysed and the minimum insert size for each group is indicated on the *x*-axis (coloured arrowheads).

We first focussed on the rRNA-mapped reads in the highly fragmented total RNA libraries. Densities of the 5′ ends of rRNA-mapped reads were calculated across all rRNAs and normalised to mRNA-mapped reads to correct for library loading. We then calculated 5′ read density in the PDD-treated sample as a percentage of that in the untreated control, to characterise zones of depletion at each probe-targeted region. Conceptually, a steep drop in 5′ read density should be seen on the right flank of the zones, starting beyond ten nucleotides upstream from the location of probe 3′ ends, as a direct consequence of DSN’s minimum requirement of ten consecutive matching base pairs for degradation [19]. Conversely, position and slope of the left flanks of the depletion zones should be determined by the distribution of insert sizes in the libraries (see schematic in Additional file 2: Figure S1A). To ascertain these patterns in our experiment we performed several *in silico* size selections, simulating libraries of different insert size ranges, and plotted their relative 5′ read densities across rRNAs. Figure 3A shows 5′ read densities in PDD-treated libraries, as a percentage of untreated, along the entire 18S rRNA. It is apparent that each probe created a zone of depletion that grew wider with increasing insert size. Overall, we saw efficient and consistent depletion for 49 of the 50 probes. The reason for failure of one probe (against 25S rRNA, position 1512) is unclear, although it was shorter than most of the others (see red ‘x’ in Figure 2B), thus it may be prudent to design probes of >22 nt length. Figure 3B displays all probe-targeted regions in 18S and 25S rRNA (except that of the failed 25S probe), aligned according to the 3′ end of each probe, to assess depletion zone topology for different library insert size ranges. Consistent across all probes we saw the expected steep drop in read density beyond ten nucleotides upstream of probe 3′ ends. Coverage recovered further upstream as a function of insert length cutoffs, with longer library inserts yielding larger depletion zones. We next explored the relationship between library insert size, inter-probe distance and read depletion. PDD should achieve continuous rRNA depletion for inter-probe distances that are at least 20 nt shorter than the minimum insert size of the library. This would allow every rRNA insert to be targeted by at least one probe (Additional file 2: Figure S1B). The results obtained from tallying reads falling between probes (Figure 3C) fully confirmed these expectations. For each insert size-range analysed, the relative depletion of inter-probe reads was greater for more closely spaced probes, approaching full efficiency as inter-probe distance dropped below the minimum insert size cutoff minus 20 nt. Assessing all inter-probe regions below this distance requirement further allowed us to calculate an average depletion efficiency of ~94% for PDD in its current configuration.

### Absence of bias in retained transcriptome coverage

**Figure 4:**
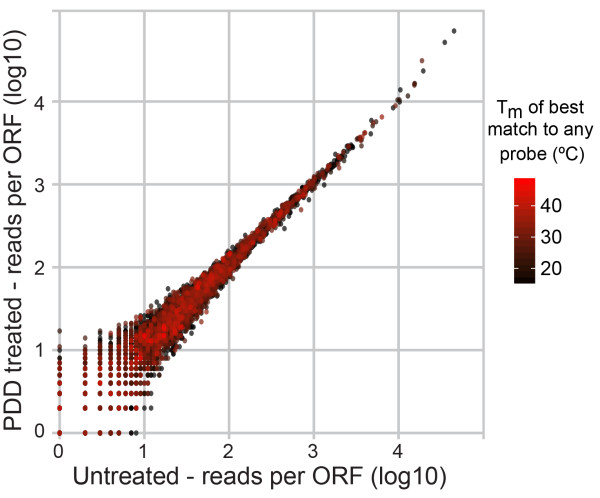
**Comparison of read counts for mRNAs from both PDD treated and untreated libraries, derived from the 20% RiboMinus™ RNA spiked sample**. Calculated T_m_ of the best off-target match within an ORF to any of the probes is indicated by the intensity of red colour.

We assessed several quality parameters in all four sequencing library datasets (Additional file 1: Table S2) and found no notable change with DSN treatment. The GC content of mRNA reads was not appreciably affected by PDD (<2% difference), unlike previously reported with C_0_T hybridization [20]. Focussing on ORF-mapped reads in the mRNA-enriched libraries, <2% of reads were PCR duplicates and little fragment bias was detected when comparing the observed read distribution to that produced by random reshuffling of reads, with or without PDD treatment (Additional file 2: Figure S2). These libraries again displayed the expected rRNA depletion pattern in the PDD-treated sample (data not shown), and were further processed to assess mRNA representation (Figure 4). This revealed high concordance between libraries and, importantly, no systematic bias as a result of the DSN treatment, even for mRNAs carrying potential off-target sites for the depletion probes (red dots) as predicted by OffTarget_Tm.

## Discussion

In this study we characterised PDD as an efficient, accurate and flexible method for the depletion of problematic sequences from RNA-seq libraries. In applying it to *S. cerevisiae* rRNA we showed that PDD performance is reliable and predictable as a function of the well characterised enzymatic properties of DSN, probe design (using our OffTarget_Tm tool) and spacing, as well as library insert size distribution. When starting with intact total RNA samples there is no need for contiguous probe coverage of the target, rather a probe spacing of 20 nt less than the minimum anticipated library insert size, or closer, will suffice to obtain even and continuous depletion. rRNA depletion by PDD in its present configuration is slightly less efficient (~94%) than that reported for the RNase H method or commercial Ribo-Zero™/RiboMinus™ kits (reportedly >99% [7, 8]). Offsetting this are other advantages of PDD such as its high stringency/lack of bias and avoidance of nuclease exposure prior to linker ligation. The relatively sparse probe spacing required further increases flexibility of probe design so that off-targets can be avoided.

Given the low cost of unmodified DNA oligonucleotides, PDD is an affordable solution for depletion of rRNA in species for which off-the-shelf commercial kits are not available. Similarly, PDD might be a cost-effective option when sequencing large sample sets derived from standard organisms. Its faithful preservation of fragment ends makes it highly suitable for applications that rely heavily on accurate mapping of fragment ends, such as ribosome profiling, CLIP-seq and related approaches, mapping of transcript extremities, and nucleotide-resolution mapping of base modification in RNA (e.g. by aniline-mediated RNA cleavage) [21, 22]. As PDD operates at the cDNA level it could also be applied to remediate pre-existing libraries containing unacceptable levels of unwanted sequences (e.g. rRNA after failed depletion attempts with the source RNA sample) or to remove “adapter dimers” (by targeting adapter-adapter junctions).

We implemented PDD in combination with the split adapter method, however, it should be applicable to most directional RNA-seq library preparation methods, with some modifications, if it is performed after linker ligation and reverse transcription but before amplification (although some precautionary measures, e.g. linker decoys or modified linkers, may be necessary if one or both linkers are double-stranded). We have tested 1× DSN buffer for hybridization and DSN digestion. While buffers that are similar in terms of ionic strength and pH should also permit PDD, any free divalent cations other than Mg^+2^ (e.g. Mn^+2^ such as is used in the CircLigase™ buffer) should be removed or chelated with equimolar EDTA (which binds Mn^+2^ preferentially to Mg^+2^) as they are known to alter DSN activity [23] and interfere with fidelity of a related dsDNA nuclease [24]. Note that it is important to eliminate the template RNA prior to PDD as DSN can also degrade the DNA strand of RNA:DNA hybrids longer than 15 nt [23]. Conversely, this property of DSN might even be harnessed productively, by using purified fragmented rRNA instead of oligonucleotides to direct DSN cleavage.

Additional potential applications and variations of the PDD procedure are numerous. Identification of minor organisms present in mixed environmental samples (e.g. for microbial transcriptomics) might also be aided by PDD, through selective depletion of conserved rRNA loci, allowing deeper sequencing of more variable rRNA regions for discriminating between species. The rapid flexibility of PDD also allows unwanted sequences other than rRNA or adapter dimers to be readily targeted for a variety of applications. Highly abundant mRNAs in the target cell or tissue to be sequenced (e.g. globin mRNA from blood cells [25]) could be depleted. Capture-seq, a technique for focussed sequencing of rare transcripts [1], could be combined with PDD to deplete abundant transcript isoforms or contaminants, for example to find rare expressed antibody variants in immune cell populations responding to antigen. The mismatch-discrimination of DSN could potentially allow the enrichment of rare point mutation-containing sequences in other types of pooled samples [19], for example to analyse the deep mutational landscape of retrovirus in a patient’s bloodstream.

## Conclusions

We envisage that PDD will prove useful for ridding RNA-seq libraries of sequences that would otherwise dominate coverage. Key features of PDD are its relatively low cost, flexibility and accuracy, allowing it to be customised to a variety of source species and applications. In this way PDD will facilitate the ever increasing and diverse uses of RNA-seq in the modern life sciences.

## Data availability

Sequencing data is available at the NCBI SRA database (http://www.ncbi.nlm.nih.gov/sra) under project accession number: SRP041813.

## Electronic supplementary material

Additional file 1: Table S1: Is a list of oligonucleotides used in this study. **Table S2**. Is a summary of diagnostic statistics for sequencing quality from the sequencing run. (DOCX 23 KB)

Additional file 2: Figure S1A and S1B: Are schematic diagrams describing expected 5′ read densities after PDD treatment, and **Figure S2**. shows an analysis of PCR-duplication in the sequencing data. (DOCX 372 KB)

Additional file 3: **Contains a Perl script, Offtarget_tm.pl that was used to estimate matches between probes and transcripts in an annotated genome**. (ZIP 9 KB)
