# Supplementary material for: Selective and flexible depletion of problematic sequences from RNA-seq libraries at the cDNA stage
Source: BMC Genomics. 2014 May 26;15(1):401. doi: 10.1186/1471-2164-15-401 (PMC4045971; doi:10.1186/1471-2164-15-401)
Supplement: Supplementary file 1 — Additional file 1: Table S1: Is a list of oligonucleotides used in this study. Table S2. Is a summary of diagnostic statistics for sequencing quality from the sequencing run. (DOCX 23 KB) [file 12864_2014_6092_MOESM1_ESM.docx]

| **Name** | **Target** | | **Location** | | **Sequence 5'-3'** |
| --- | --- | --- | --- | --- | --- |
| **rRNA Depletion probes:** | | | | | |
| 18S-1 | 18S rRNA | | | 4 | CTGGTTGATCCTGCCAGTAGTCATATGCTTGTC |
| 18S-2 | 18S rRNA | | | 38 | CAAAGATTAAGCCATGCATGTCTAAGTATAAGC |
| 18S-3 | 18S rRNA | | | 79 | CAGTGAAACTGCGAATGGCTCATTAAATCAG |
| 18S-4 | 18S rRNA | | | 139 | CATGGTATAACTGTGGTAATTCTAGAGCTAATACATGC |
| 18S-5 | 18S rRNA | | | 194 | TGGAAGAGATGTATTTATTAGAT |
| 18S-6 | 18S rRNA | | | 242 | TGATGATTCATAATAACTTTTCGAATCGCATGGC |
| 18S-7 | 18S rRNA | | | 283 | TGGCGATGGTTCATTCAAATTTCTGCCCTATCAACTT |
| 18S-8 | 18S rRNA | | | 447 | TCCTAATTCAGGGAGGTAGTGACAATAAATAACGA |
| 18S-9 | 18S rRNA | | | 528 | TACCTTAACGAGGAACAATTGGAGGGCAAGTC |
| 18S-10 | 18S rRNA | | | 551 | GGGCAAGTCTGGTGCCAGCAGCCGCGGTAATTCCAGCTCC |
| 18S-11 | 18S rRNA | | | 680 | TTCCAACGGGGCCTTT |
| 18S-12 | 18S rRNA | | | 749 | TTGAAAAAATTAGAGTGTTCAAAGCAGGCGT |
| 18S-13 | 18S rRNA | | | 781 | TTGCTCGAATATATTAGCATGGAATAATAGAAT |
| 18S-14 | 18S rRNA | | | 856 | ATGATTAATAGGGACGGTCGGGGGCAT |
| 18S-15 | 18S rRNA | | | 947 | TGCCAAGGACGTTTTCATTAATCAAGAACGA |
| 18S-16 | 18S rRNA | | | 1027 | ACTATGCCGACTAGGGATCGGGTGGTGTT |
| 18S-17 | 18S rRNA | | | 1066 | CCCACTCGGCACCTTACGAGAAATCAAAGTCTT |
| 18S-18 | 18S rRNA | | | 1211 | AGGTCCAGACACAATAAGGATTGACAGAT |
| 18S-19 | 18S rRNA | | | 1287 | AGTTGGTGGAGTGATTTGT |
| 18S-20 | 18S rRNA | | | 1465 | CGCTACACTGACGGAGCCAGCGAGT |
| 18S-21 | 18S rRNA | | | 1654 | GATTGAATGGCTTAGTGAGGCCTCAGGATC |
| 18S-22 | 18S rRNA | | | 1714 | AGCGGAGAATTTGGACAAACTTGGTCATT |
| 18S-23 | 18S rRNA | | | 1782 | ACCTGCGGAAGGATCATTA |
| 18S-24 | 18S rRNA* | | | 1782 | ACCTGCGGAAGGATCATTAAAAAAA |
| 25S-1 | 25S rRNA | | | 5 | GACCTCAAATCAGGTAGGAGTACCCGCTGAACT |
| 25S-2 | 25S rRNA | | | 58 | GGAAAAGAAACCAACCGGGATTGCCTTAG |
| 25S-3 | 25S rRNA | | | 193 | CTTGGAACAGGACGTCATAGAGGGTGAGAATC |
| 25S-4 | 25S rRNA | | | 266 | AGAGTCGAGTTGTTTGGGAATGCAGCTCTAAGT |
| 25S-5 | 25S rRNA | | | 356 | CAGTGATGGAAAGATGAAAAGAACTTTGAAAAG |
| 25S-6 | 25S rRNA | | | 495 | GCCAGCATCAGTTTTGGTGGCAGGAT |
| 25S-7 | 25S rRNA | | | 569 | AATACTGCCAGCTGGGACTGAGGACTG |
| 25S-8 | 25S rRNA | | | 662 | TCTAACGTCTATGCGAGTGTTTGGGTGTAAAAC |
| 25S-9 | 25S rRNA | | | 734 | CAAGAGGTGCACAATCGACCGATCCTGA |
| 25S-10 | 25S rRNA | | | 921 | ATCGAACCATCTAGTAGCTGGTTCCTGCCGAAGT |
| 25S-11 | 25S rRNA | | | 1159 | ACGTAGAGTTAAGGTGCCGGAATACACGCTC |
| 25S-12 | 25S rRNA** | | | 1513 | GGACGTGGGTTAGTCGATC |
| 25S-13 | 25S rRNA | | | 1623 | GGATTCTTCACGGTAACGTAACTGAATG |
| 25S-14 | 25S rRNA | | | 1698 | CAGCTTATCACCCCGGAATTGGTTTA |
| 25S-15 | 25S rRNA | | | 1725 | CCGGAGATGGGGTCTT |
| 25S-16 | 25S rRNA | | | 1780 | GCTTGTGACGGCCCGTGAAAATCCACAGGAAGG |
| 25S-17 | 25S rRNA | | | 1801 | TCCACAGGAAGGAATAGTTTTCATGCCAGGTCGTAC |
| 25S-18 | 25S rRNA | | | 2211 | TCAAAGTGAAGAAATTCAACCAAGCGCGGGTAA |
| 25S-19 | 25S rRNA | | | 2448 | GAGGGTGTAGAATAAGTGGGAGCTTCGGCGC |
| 25S-20 | 25S rRNA | | | 2546 | CACGTTCTAGCATTCAAGGTCCC |
| 25S-21 | 25S rRNA | | | 2890 | ATACCGAAGCAGAATTCGGTAAGCGTTGGATTGT |
| 25S-22 | 25S rRNA | | | 3173 | GATGGATACGAATAAGGCGTC |
| 25S-23 | 25S rRNA | | | 3259 | TGCTGGCGAATTGCAATGTC |
| 25S-24 | 25S rRNA | | | 3295 | AATCATTTGTATACGACTTAGATGTACAACGGG |
| 5.8S-1 | 5.8S rRNA | | | 135 | GGGCATGCCTGTTTGAGCGTCATT |
| 5S-1 | 5.8S rRNA | | | 98 | ACGCGAAACTCAGGTGCTGCAATCT |
| **Name** | **Target** | **Location** | | | **Sequence 5'-3'** |
| **qPCR primers:** | | | | | |
| CPR6-F | CPR6 | | 481 | | ACAAGCCATTGCGTGATGTA |
| CPR6-R | CPR6 | | 554 | | GGCATTCTCTGGCACTTGAT |
| SSC1-F | SSC1 | | 914 | | CCTGCCATTTATCACTGCTG |
| SSC1-R | SSC1 | | 980 | | TGAGCCCTGGAGAACTTCAT |
| 18S-F1 | 18S rRNA | | 726 | | CTTGGCGAACCAGGACTTT |
| 18S-R1 | 18S rRNA | | 779-R | | ACGCCTGCTTTGAACACTCT |
| 18S-F2 | 18S rRNA | | 984 | | GGGGATCGAAGATGATCAGA |
| 18S-R2 | 18S rRNA | | 1045-R | | CGATCCCTAGTCGGCATAGT |
| 25S-F3 | 25S rRNA | | 1292 | | CTAGCCCTGAAAATGGATGG |
| 25S-R3 | 25S rRNA | | 1396-R | | GCTTCGTCACTGACCTCCAC |
| **Reverse transcription primer:** | | | | | |
| TVN-RT | n/a | | n/a | | 5'Phos-GATCGTCGGACTGTAGAACTCTGAACG  /iSp9/GTGACTGGAGTTCCTTGGCACCCGAGAA  TTCCATTTTTTTTTTTTTTTTTTTTVN-3' |

* Probe includes part of poly(A) tract

** Probe failed to deplete target

**Table S1.** Oligonucleotide sequences used in this study.

| **RiboMinus™ RNA : total RNA mix (1:4); fragmented 4 min @ 94°C** | **Untreated** | **PDD-treated** |
| --- | --- | --- |
| Total reads | 20.0M | 16.0M |
| Too-short / p(A) reads | 1.64M | 2.71M |
| GC content of mRNA-mapped reads | 41.7% | 42.6% |
| PCR duplication rate (% of reads) | 1.56% | 1.79% |
| % of filtered reads mapped | 86.0% | 82.9% |
| **Total RNA; fragmented**  **8 min @ 94°C** | **Untreated** | **PDD-treated** |
| Total reads | 18.4M | 7.8M |
| Too-short / p(A) reads | 1.37M | 1.46M |
| GC content of mRNA-mapped reads | 39.1% | 37.1% |
| % of filtered reads mapped | 95.0% | 91.6% |

**Table S2.** PCR duplication rate was calculated from the duplication rate in ORFs with <2% coverage and 2 or more reads (Figure S2). Coverage is defined as 5' read-ends divided by the ORF area available for mapping (ORF length minus median library insert length). Reads shorter than 11 nt after trimming low quality (phred score <28), poly(A), or adapter sequences were discarded. Mapping against the current yeast transcriptome was performed using Bowtie2, prioritizing rRNA transcripts as described in Methods.
