# Supplementary material for: Selective and flexible depletion of problematic sequences from RNA-seq libraries at the cDNA stage
Source: BMC Genomics. 2014 May 26;15(1):401. doi: 10.1186/1471-2164-15-401 (PMC4045971; doi:10.1186/1471-2164-15-401)
Supplement: Supplementary file 2 — Additional file 2: Figure S1A and S1B: Are schematic diagrams describing expected 5′ read densities after PDD treatment, and Figure S2. shows an analysis of PCR-duplication in the sequencing data. (DOCX 372 KB) [file 12864_2014_6092_MOESM2_ESM.docx]

**
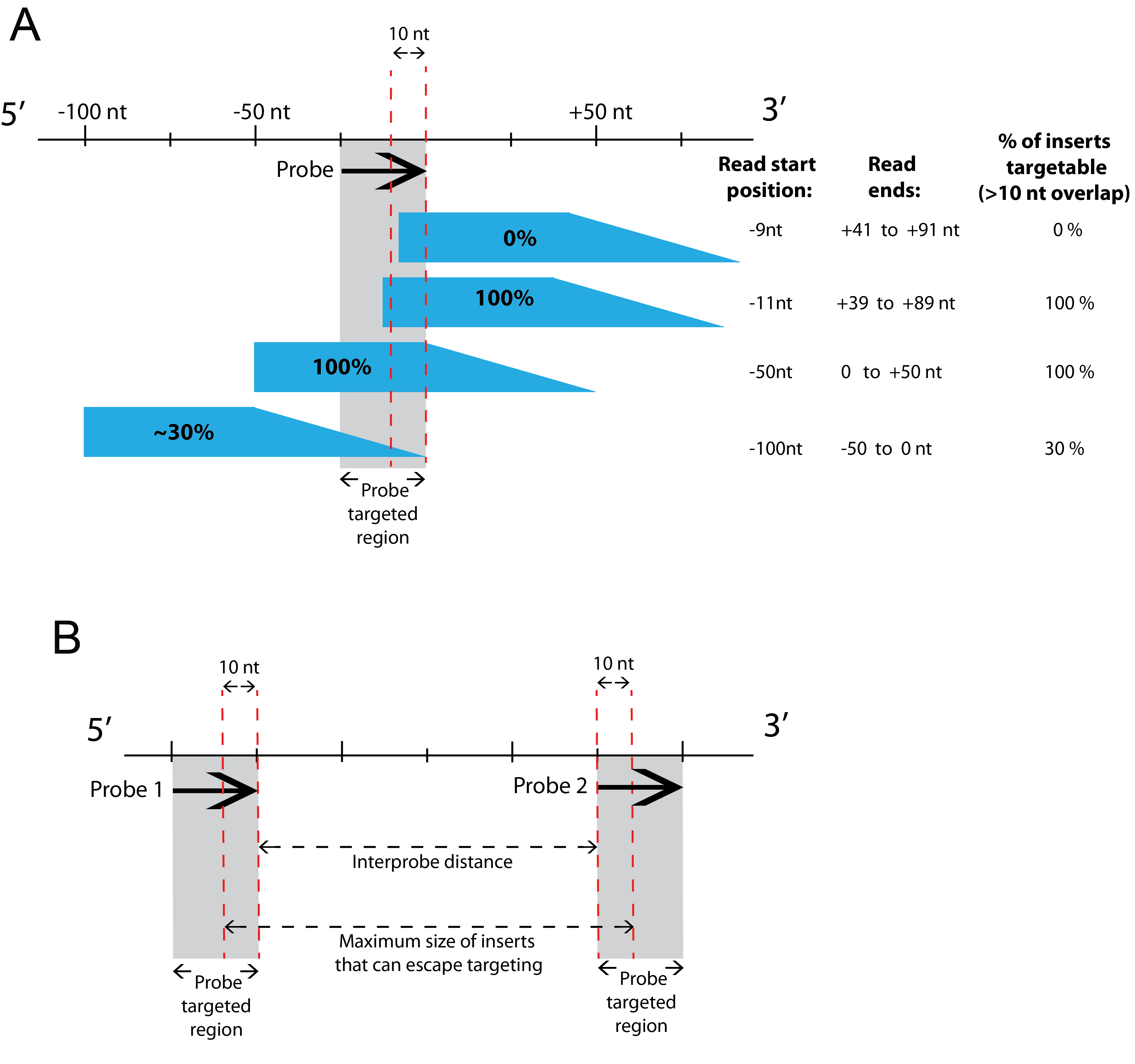
**

**Figure S1.** **A.** The expected depletion pattern observed around a probe binding site in an idealized RNA-seq library with insert sizes ranging from 50 nt to 100 nt. Blue bars: coverage distribution of library inserts for particular 5' read locations. Coordinates are relative to the 3' end of the probe binding site (dashed red line). At least 10 nt of overlap between the insert and probe is required for target degradation by DSN. **B.** Schematic of two adjacent probes, showing the minimum 10 nt overlap between an intervening insert and the probes, which is required for DSN to digest the insert.


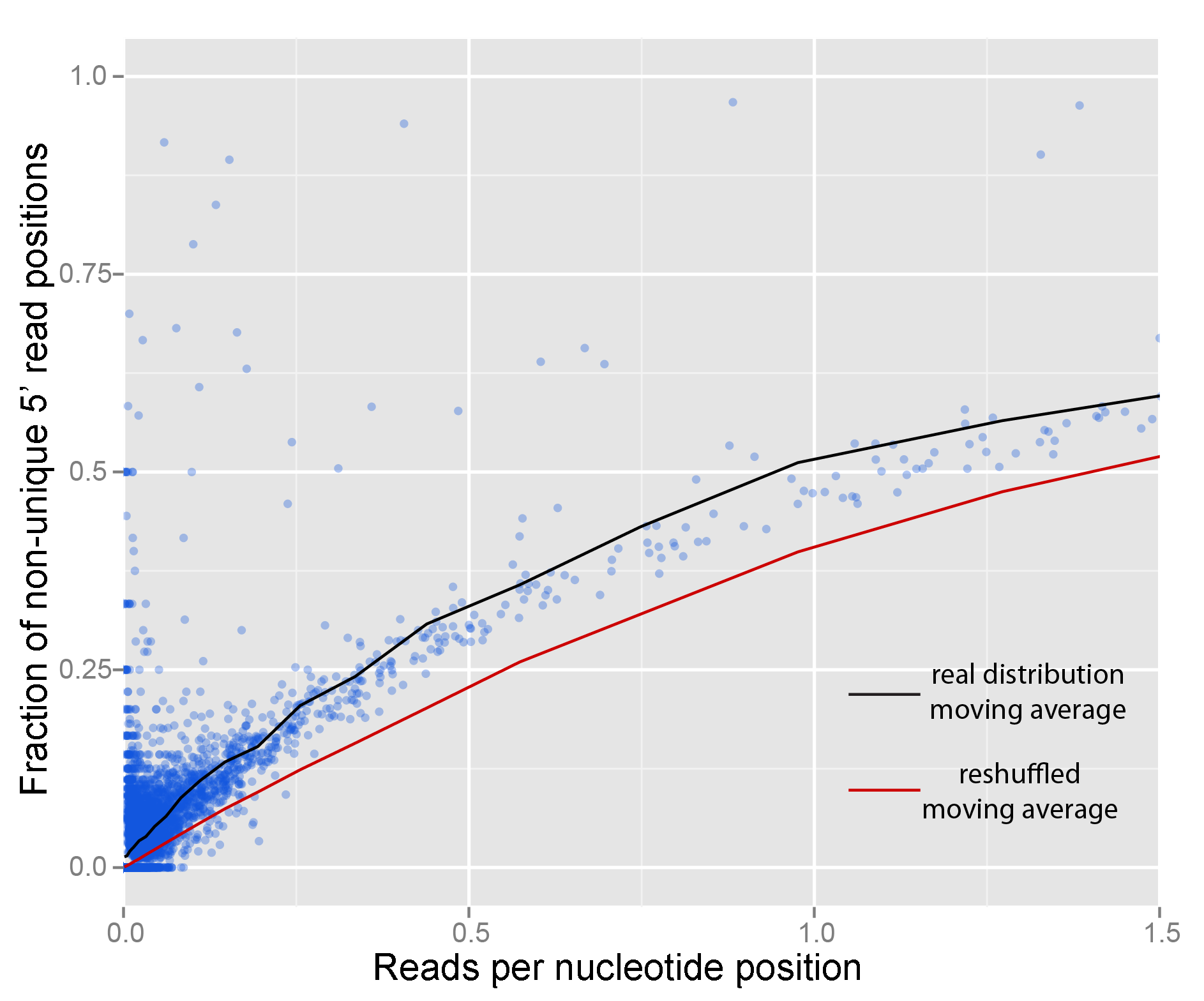


**Figure S2.**

Library PCR duplicate levels were estimated by comparing the fraction of non-unique 5' fragment ends for each yeast ORF with two or more reads (blue dots, black line) to that expected by chance (red line) from simulations where reads were randomly re-located within each ORF. As the coverage level (reads per nucleotide position) approaches zero, the real number of duplicates approaches <2% (y-intercept of black line) while the theoretical distribution approaches zero. Shown is the data from non-PDD treated, RiboMinus™-spiked RNA library (a similar curve was obtained from the PDD-treated library, see Additional file 2: Table 2). The greater level of duplication in more highly covered transcripts indicates the presence of some bias in fragment end location rather than PCR duplication.
